# Method for the Evaluation of the Homogeneity of Asphalt Mixtures by 2-Dimensional Image Analysis

**DOI:** 10.3390/ma15124265

**Published:** 2022-06-16

**Authors:** Pei Sun, Ke Zhang, Sen Han, Zijun Liang, Wei Kong, Xuejuan Zhan

**Affiliations:** 1 School of Urban Construction and Transportation, Hefei University, Hefei 230601, China; lzj@hfuu.edu.cn (Z.L.); kongweismile@163.com (W.K.); zxj2710906692@163.com (X.Z.); 2 College of Information Engineering, Fuyang Normal University, Fuyang 236041, China; zhangke_365@126.com; 3 Highway and Airport Pavement Research Center, School of Highway, Chang’an University, Xi’an 710064, China; hyram_hs@chd.edu.cn; 4 Anhui Province Transportation Big Data Analysis and Application Engineering Laboratory, Hefei University, Hefei 230601, China

**Keywords:** asphalt mixture, digital image processing, evaluation index, stereology theory, homogeneity

## Abstract

In order to evaluate the homogeneity of asphalt mixture quantitatively, the distribution characteristic of internal phases of asphalt mixture were identified based on digital image processing technique and stereology theory, and the homogeneity coefficient (i.e., *K*) was proposed. At the same time, the trend of variation and reliability of homogeneity of asphalt mixture were analyzed by changing the nominal maximum aggregate size, aggregate gradation and asphalt content. The results suggest that the homogeneity of asphalt mixture could be comprehensively described using DIP technique combined with stereology theory. The smaller the *K*, the better the distribution homogeneity of the asphalt mixture. An improvement in the homogeneity of an asphalt mixture is achieved with the decrease of the nominal maximum aggregate size and a finer aggregate gradation. The asphalt content corresponding to the optimal homogeneity of the internal structure of asphalt mixture specimen is the optimum asphalt content. According to the experimental study, the suggested values of the homogeneity coefficient were given, which provides theoretical support to control the construction quality of the hot mixture asphalt.

## 1. Introduction

The inhomogeneity of asphalt mixture is an important reason for the early damage of a pavement. It is directly related to the fatigue life, rutting performance, moisture damage and tensile strength, and especially to the service life of the pavement [1,2,3,4,5,6]. Previous studies have shown that in coarse-aggregate-rich regions, water was easily able to penetrate into the interior of asphalt mixture due to the larger volume of air voids, which was extremely unfavorable to the water stability of asphalt mixture. In fine-aggregate-rich regions, meanwhile, the rutting resistance and skid resistance of asphalt mixture were significantly reduced [7,8]. It has long been recognized that the homogeneity of asphalt mixture is closely related to pavement performance, but very little research has been conducted to explore the homogeneity of laboratory-compacted asphalt mixture specimens due to the difficulty in characterizing the homogeneity using traditional testing methods [9,10].

In recent years, digital image processing (i.e., DIP) technique has developed rapidly in the field of road engineering and has been applied to the quantitative evaluation of the homogeneity of asphalt mixture [11,12,13,14,15]. Different from the traditional method of indirectly evaluating the homogeneity of mixture by density of asphalt mixture, percent air voids and texture depth, the homogeneity of asphalt mixture can be directly characterized by its morphological characteristics using digital image processing technique.

Hunter et al. proposed the index of variation degree of the cumulative area of aggregate particles in the section image to describe the homogeneity of asphalt mixture [16]. Wu et al. selected red stones instead of general coarse aggregates and used the distribution homogeneity of coarse aggregate particles to characterize the internal homogeneity of asphalt mixture [17]. Thyagarajan et al. proposed two new indexes (i.e., HIver and HIlat) to characterize air voids distribution. The smaller the HIver and HIlat, the better the homogeneity of an asphalt mixture [18]. Zhang et al. reconstructed 3-dimensional gradation characteristics of asphalt mixture specimens and proposed to use the variation of inertia of the X-axis and the statistical index of the aggregate centroids to evaluate the horizontal distribution homogeneity and the vertical distribution homogeneity of asphalt mixture respectively [19]. It was found that the virgin aggregate and the new aggregate in the recycling asphalt mixture could be identified using DIP technique, and the homogeneity coefficient proposed based on the aggregate particle area ratio and the deviation degree of the new aggregate particles could characterize the homogeneity of recycling asphalt mixture [20]. Moreover, the quantity distribution and location distribution of coarse aggregates could characterize the homogeneity of the mixture effectively [21,22,23,24].

Previous studies show that great progress has been made in the research related to the homogeneity of the asphalt mixture. However, the existing research mainly focuses on using the characteristic parameters of aggregates to evaluate the homogeneity of asphalt mixtures, and the role of other components (i.e., air voids and asphalt mortar) in the internal homogeneity of the asphalt mixture is rarely studied. In addition, if the optimized design of asphalt mixture based on homogeneity can be realized, the internal homogeneity of asphalt mixture can be improved effectively from the design stage, thus extending the service life of asphalt pavement. However, very little research has been conducted to explore the effect of design factors on the homogeneity of asphalt mixture from the micro-level.

Exploring the relationship between the homogeneity of asphalt mixture and the design factors can provide a theoretical basis for the design of asphalt mixture based on homogeneity. The primary objective of this study is to develop a method for evaluating the homogeneity of asphalt mixture quantitatively using DIP technique. The design factors on the homogeneity of asphalt mixture will also be analyzed. This study provides theoretical support to control the construction quality of the hot mixture asphalt.

## 2. Experimental Study

### 2.1. Materials

High-viscosity SBS (i.e., styrene-butadiene-styrene) modified asphalt was used for open-graded asphalt friction course (i.e., OGFC) mixture, while SBS modified asphalt was used for asphalt concrete (i.e., AC) mixture and stone mastic asphalt (i.e., SMA) mixture. The properties of the asphalt binders are shown in Table 1. In order to better extract aggregate particles in the subsequent image preprocessing process, limestone with lighter color was selected, and the technical properties met the requirements of the specification [25]. In the preparation of SMA mixture, the added lignin fiber content was 0.3% of the mass of the asphalt mixture.

### 2.2. Experiment Design

Although the homogeneity of asphalt mixture is directly related to the pavement performance, it is not considered in the current design method of asphalt mixture. In the design process of asphalt mixture, aggregate gradation, asphalt-aggregate ratio and nominal aggregate maximum size would affect the homogeneity of asphalt mixture. Therefore, different asphalt mixtures were designed by changing the nominal maximum aggregate size, aggregate gradation and asphalt content, as listed in Table 2. The aggregate gradations are shown in Table 3.

### 2.3. Imaging of Cross Section

#### 2.3.1. Specimen Preparation

**Figure 1:**
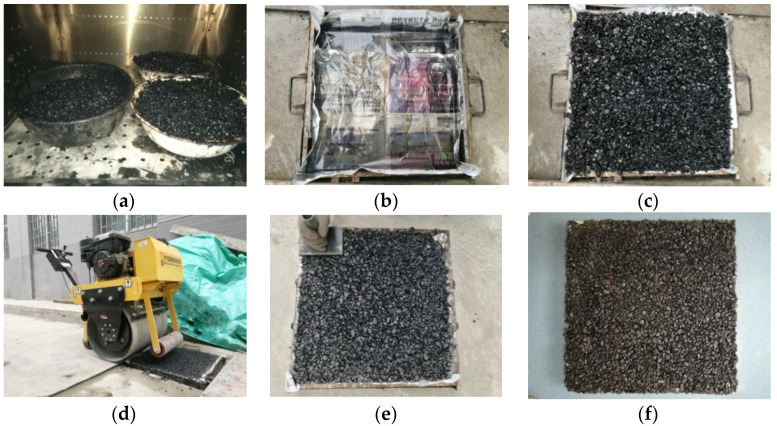
Preparation of asphalt mixture specimens with the dimensions of 500 mm × 500 mm × 50 mm: (**a**) mixed asphalt mixture; (**b**) specimen mold; (**c**) add asphalt mixture to the mold; (**d**) compaction process; (**e**) corner handling; (**f**) asphalt mixture specimen.

In order to simulate the actual pavement better, a hand-held roller compactor was used in this study to prepare asphalt mixture samples with a size of 500 mm × 500 mm × 50 mm. The width of roller steel wheel is ≥600 mm, while 600 mm includes the 500 mm sample mode width and 100 mm spare width. The roller compactor can provide 300 N/cm line compressive stress. To make the compacting degree of specimen achieve 100 ± 1% of the Marshall standard compaction density, as outlined in the method of JTG E20-2011 [26], the compaction times were determined to be 24 round trips. The compaction temperature was controlled at 175 °C during compaction, and the compaction process is shown in Figure 1.

#### 2.3.2. Cutting Prepared Specimens

Stereology theory extracts effective information from many 2-dimensional section images and makes statistical analysis of 3-dimensional structure through the mentioned 2-dimensional information [27]. Therefore, the homogeneity parameters of the asphalt mixture obtained by 2-dimensional section images can be used to assess the actual 3-dimensional homogeneity of the asphalt mixture. The following assumptions should be met firstly.

(i) Random: the selected section is independent and random.
(ii) Representative: asphalt mixture is a kind of heterogeneous material, so it is necessary to select the section that can represent the internal characteristics of the asphalt mixture to the greatest extent.
(iii) Isotropic: Asphalt mixture section images should be selected in the same direction. For example, all sections of the specimen are perpendicular to or parallel to the compaction direction.

Sefidmazgi pointed out that the more 2-dimensional sections selected, the better the 2-dimensional internal structure parameters can reflect the actual 3-dimensional internal structure of the asphalt mixture [28]. For the specimen compacted by Superpave gyratory compaction method, when the number of section images selected was six, the internal structure evaluation indexes obtained were basically the same as those obtained when the number of section images was larger.

**Figure 2:**
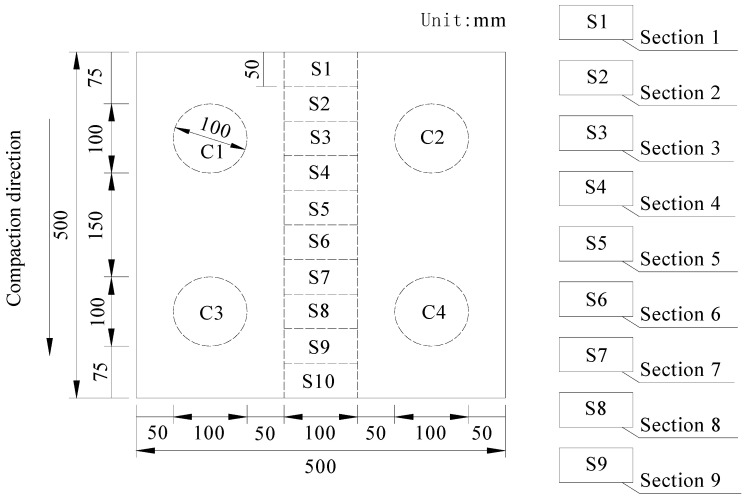
Preparation of asphalt mixture specimens.

Based on the three assumptions of stereology theory and the research result of Sefidmazgi, nine section images were acquired from each of the specimens prepared by hand-held roller compactor, and only the section surface on the same side was selected. A diamond saw was used to cut the samples with the size of 500 mm × 500 mm × 50 mm, and nine specimens with dimensions of 100 mm × 50 mm × 50 mm were obtained, labeled as S1–S9 in Figure 2. These specimens were used to test the evaluation index for the homogeneity of asphalt mixture. In addition, the cylinder core specimens with the diameter of 100 mm (i.e., C1, C2, C3 and C4 in Figure 2) were used to test the air voids of asphalt mixture.

#### 2.3.3. Sectional Image Acquisition

**Figure 3:**
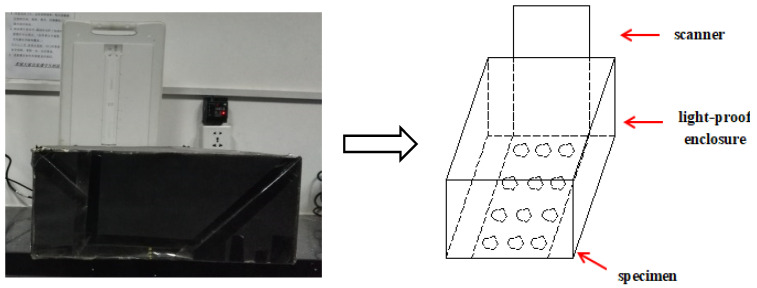
Section image acquisition.

**Figure 4:**
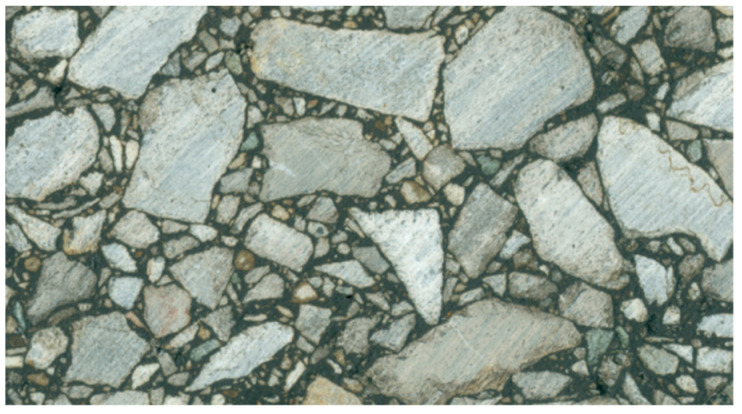
Mixture section image.

A flatbed scanner with a resolution of 1200 pix/inch was used to obtain section images of asphalt mixture specimens. Different external light sources would lead to distortion of the collected image, so a light-proof enclosure was used to cover the scanner to reduce the error caused by external light sources, as shown in Figure 3. The obtained specimen section image was shown in Figure 4.

## 3. Homogeneity Evaluation Parameters of Asphalt Mixture

### 3.1. Image Processing

**Figure 5:**
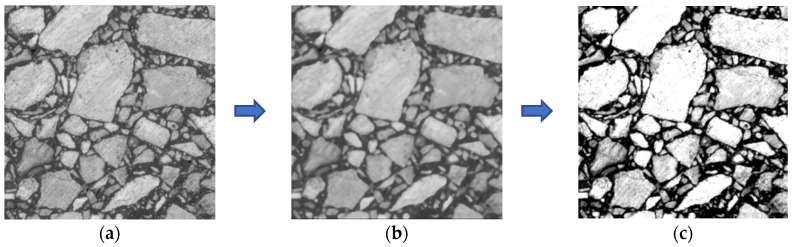
Image processing I: (**a**) gray image; (**b**) after applying median filter; (**c**) after the gray-scale transformation.

Firstly, the original image acquired by the scanner was grayed with the rgb2gray function in MATLAB, as shown in Figure 5a. In view of the fact that median filtering could better retain irregular edge information of the image while filtering out noise, median filtering was selected to eliminate noise generated in the process of image acquisition, as shown in Figure 5b. In order to distinguish asphalt mastic, air voids and aggregate better, gray-scale transformation was used to enhance the contrast of the aggregate and the asphalt mastic, as shown in Figure 5c.

**Figure 6:**
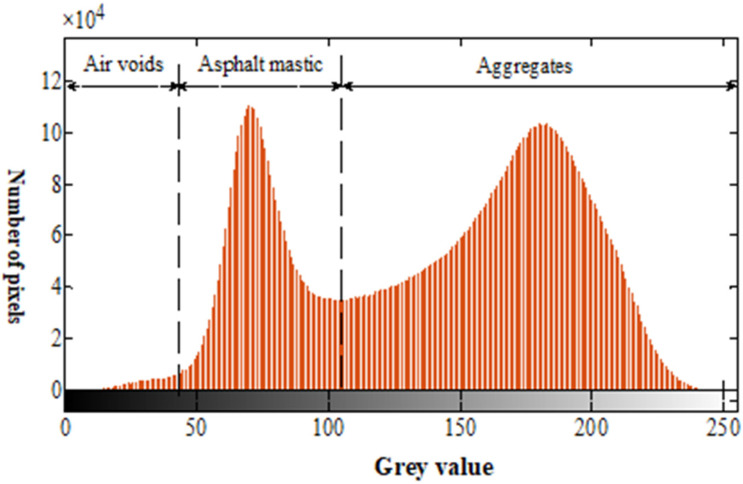
Gray histogram of asphalt mixture.

**Figure 7:**
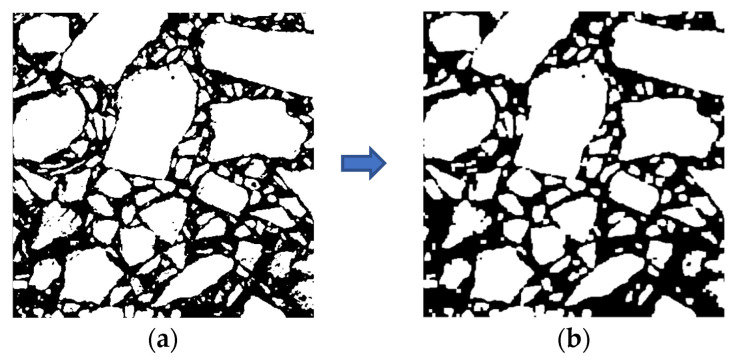
Image processing II: (**a**) OTSU; (**b**) after opening operation and closing operation.

As can be seen from Figure 5a, asphalt mastic and air voids were close to black, and aggregate particles appeared gray and white. The gray histogram of asphalt mixture had a very obvious bimodal characteristic, as shown in Figure 6. The region to the right of the axle represented aggregate particles, corresponding to a larger gray value; thus, the threshold method could be used to segment the image. After thresholding, the image was segmented into two parts: the aggregate part and the other part, while the other part included asphalt mastic and air voids. Considering that the maximum inter-class variance method (i.e., OTSU) was time-saving and accurate [29], the maximum inter-class variance method was adopted to segment the section image of asphalt mixture, as shown in Figure 7a. After the above treatment, it could be seen that although most aggregate particles were separated, some aggregate particles were still not completely separated, and a few aggregate particles exhibited the “pockhole” phenomenon. In order to eliminate the phenomenon of aggregate adhesion and local holes, opening operation was performed to break the narrow connection, and closing operation was adopted to fill the tiny holes, as shown in Figure 7b.

### 3.2. Aggregate Particle Size Distribution through Image Processing

At present, there is no standard to judge whether the selected parameters were appropriate or not in the choice of median filter value and opening-and-closing operation value during the image preprocessing process, and it mainly depends on subjective judgment. If the accuracy of image preprocessing process could not be guaranteed, it would mean that the subsequent analysis was problematic and the test results were inaccurate. This paper proposed to ensure the rationality of image preprocessing process by comparing the two-dimensional virtual gradation and laboratory gradation.

#### 3.2.1. Minimum Size of Aggregate Identifiable in the Image

Previous studies showed that the shape of aggregate particles was closest to an ellipse, so the minor axis of equivalent ellipse was used to characterize the aggregate particle size [30]. The area of the equivalent ellipse was equal to the aggregate particle area, and the major axis of the ellipse was equal to the aggregate principal axis, which referred to the farthest distance between two points on the boundary of aggregate particles.

(1) $$L_{i}=\frac{4S_{i}}{\pi B_{i}}=\frac{4S_{i}}{\pi D_{i}}$$

Here, *L_i_* and B*_i_* represent the equivalent minor axis and equivalent major axis of *i*^th^ aggregate in the image, respectively, and *S_i_* refers to the area of *i*^th^ aggregate in the image, while D*_i_* is the aggregate principal axis.

Restricted by the limited resolution of image, it was difficult to identify the aggregate particles that were too small, and the distribution characteristics of these components had little influence on the internal structure distribution of the asphalt mixture. Therefore, the composition of the asphalt mixture was simplified in this paper, including: (i) air voids, (ii) asphalt mastic, (iii) identifiable aggregate particles. A previous study showed that the minimum size of aggregate identifiable in the image was approximately 10–20 times the resolution of the image [31]. Since the resolution selected in this paper was 1200 pix/inch (i.e., 0.021 mm/pix), the minimum identifiable aggregate size (i.e., *L*_min_) in the image was 0.6 mm. In subsequent discussions, aggregate particles smaller than 0.6 mm were no longer considered.

#### 3.2.2. Image-Based Aggregate Gradation (i.e., Virtual Gradation)

The total volume of an asphalt mixture is composed of aggregate, air voids and asphalt mastic.

(2) $$V_{\mathrm{mix}}=V_{a}+V_{m}+V_{v}$$

Here, *V*_mix_ is the volume of asphalt mixture, *V_a_* is the volume of aggregate, *V_m_* and *V_v_* are the volume of asphalt mastic and air voids, respectively.

From the aggregate gradation listed in Figure 1, the quantity of aggregate particles of different size required to prepare an asphalt mixture specimen could be calculated. Meanwhile, because the relative densities of aggregate particles of different sizes were known, the volume of aggregates of different sizes could be calculated. Then, the proportion of identifiable aggregate particle volume to the total aggregate particle volume (i.e., *IAP*) could be expressed in Equation (3).

(3) $$IAP=\frac{\sum_{i}V_{i}}{V_{a}}=\frac{V_{I}}{V_{a}},i\in L_{i}>L_{\min}$$

Here, *V_I_* is the volume of identifiable aggregates with particle size greater than *L*_min_, *V_i_* is the volume of aggregates retained on *i*^th^ sieve.

The area of aggregate particles with particle size greater than 0.06 mm in the processed section image was counted, and the total area of aggregates in the section image is determined according to Equation (6).

(4) $$A_{m}=N_{m}\cdot \Delta x^{2}$$

(5) $$A_{I}=\sum_{m}A_{m},m\in L_{m}\geq L_{\min}$$

(6) $$A_{a}=\frac{A_{I}}{IAP}$$

Here, *A_m_* is the area of the *m*^th^ aggregate whose particle size is greater than *L*_min_ in the section image, *N_m_* is the number of pixels contained in the *m*^th^ aggregate, Δx is the length of each pixel which was depended on the image resolution, *A_I_* is the area of the identifiable aggregate particles in the section image, *A_a_* is the total area of the aggregates (i.e., fine aggregate and coarse aggregate).

The percentage of aggregates retained on the *i*^th^ sieve calculated using the two-dimensional area (i.e.,$PR_{i}^{A}$) could be expressed in Equation (7).

(7) $$PR_{i}^{A}=\frac{\sum_{k=i}^{i+1}A_{k}}{A_{a}}\times 100=\frac{\sum_{k=i}^{i+1}A_{k}}{A_{I}}\times IAP\times 100\;i\in L_{i}>L_{\min},k\in L_{k}>L_{\min},L_{i}<L_{k}<L_{i+1}$$

Here, *A_k_* refers to the area of the *k*^th^ aggregate and *k* refers to the sieve size between *i* and *i* + 1.

The aggregate gradation could be obtained by the percentage of aggregates retained. The two-dimensional virtual aggregate gradation obtained by the processed image was compared with the actual laboratory aggregate gradation. The filter parameters selected in Section 3.1 may need to be adjusted repeatedly until the error between the percentage of aggregates retained on the *i*^th^ sieve acquired based on two-dimensional area (i.e.,$PR_{i}^{A}$) and the actual percentage of aggregates retained on the *i*^th^ sieve calculated by laboratory meets the specified requirement. The specified requirement is that the probability of keeping the error at [−5%, 5%] is ≥95%.

### 3.3. Homogeneity Evaluation Parameters Analysis

Asphalt mixture is a kind of multi-component composite material composed of coarse aggregate, asphalt mortar (i.e., asphalt mastic and fine aggregate) and air voids. Previous studies put forward that asphalt mortar can be distributed evenly in the mixture [19]. Therefore, in this paper, asphalt mortar is not considered when analyzing the internal structure uniformity of asphalt mixture.

#### 3.3.1. Region Division

For circular section image, the annular segmentation can reflect the distribution characteristics in the radial direction, while the sector segmentation can reflect the distribution characteristics in the annular direction [32]. Meanwhile, it should be noted that the section cannot be divided at will. If the area of each region is too large, the homogeneity of the asphalt mixture cannot be characterized accurately. If divided regions are too small, it is easy to divide the aggregate with larger particle size into several aggregates with smaller particle size, leading to greater errors and affecting the reliability of the calculation results.

**Figure 8:**
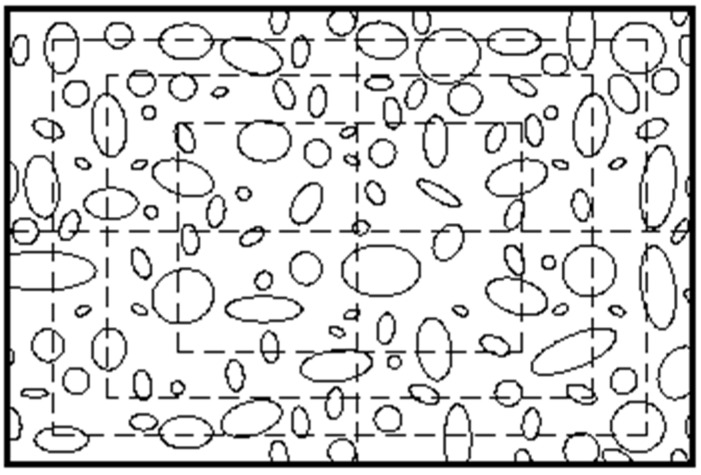
Divided method of section image.

Considering the advantages of the two divided methods and the influence of aggregate particle size, the section image of asphalt mixture specimen was divided into 16 regions with equal area, as shown in Figure 8. This divided method cannot only describe the distribution characteristics in angle region, but also reflect the distribution characteristics from the radial direction.

#### 3.3.2. Evaluation Parameter of Aggregate Distribution Homogeneity

For homogeneous asphalt mixture specimens, the total area of coarse aggregate particles contained in the section of the same area are equal. The particle area was taken as the evaluation parameter of aggregate distribution characteristic in each region of the image, and the variation coefficient of the sum of coarse aggregate particle area in each region could be calculated according to Equation (9).

(8) $$\bar{A}=\frac{1}{n}\sum_{i=1}^{4}\sum_{j=1}^{4}A_{ij}$$

(9) $$k_{a}==\frac{\sqrt{\frac{1}{n-1}\sum_{i=1}^{4}\sum_{j=1}^{4}{\left(A_{ij}-\bar{A}\right)}^{2}}}{\bar{A}}$$

Here, *n* indicates the number of regions divided, $\bar{A}$ is average area of coarse aggregates in the section image; *k*_a_ is the variation coefficient of aggregate particle area. The smaller the *k*_a_, the more uniform the aggregate particle distribution in this section image.

#### 3.3.3. Evaluation Parameter of Air Voids Distribution Homogeneity

**Figure 9:**
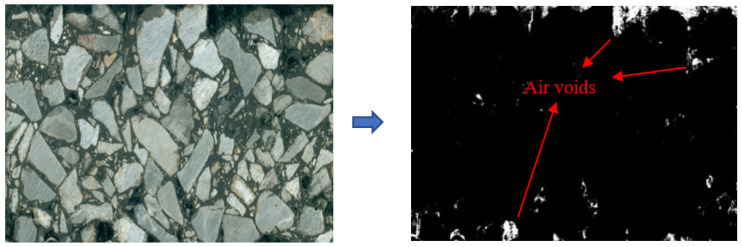
Air voids extraction process.

In the section image of asphalt mixture specimen, the order of the darkness from dark to light was: air voids, asphalt mortar (i.e., fine aggregate, asphalt and filler), coarse aggregate. Image Pro-Plus (IPP) can distinguish different mix components by setting different color ranges in advance and allows the thresholds for each color range to be adjusted, while the thresholds are automatically selected by the computer. Considering the advantages of IPP software, this paper uses the software to distinguish air voids from other components of the mixture and calculate the area of the air voids. Figure 9 shows the marking and extraction process of air voids.

We counted the area of air voids in each region and the variation coefficient of the air voids’ area was used as a parameter to evaluate the distribution homogeneity of air voids in the section image, as shown in Equation (11).

(10) $$\bar{V}=\frac{1}{n}\sum_{i=1}^{4}\sum_{j=1}^{4}V_{ij}$$

(11) $$k_{v}==\frac{\sqrt{\frac{1}{n-1}\sum_{i=1}^{4}\sum_{j=1}^{4}{\left(V_{ij}-\bar{V}\right)}^{2}}}{\bar{V}}$$

Here, *n* indicates the number of regions divided, $\bar{V}$ is average area of air voids in the section image; *k_v_* is the variation coefficient of air voids area.

#### 3.3.4. Evaluation Index of Asphalt Mixture Homogeneity

Compared with air voids, the coarse aggregate area in the section image was much larger, so weight coefficients (i.e., α_1_ and α_2_) were introduced.

(12) $$\alpha_{1}=S_{C}/(S_{V}+S_{C})$$

(13) $$\alpha_{2}=S_{V}/(S_{V}+S_{C})$$

(14) $$k=\alpha_{1}*k_{a}+\alpha_{2}*k_{v}$$

where *S_C_* and *S_V_* are the total area of coarse aggregate particles (equivalent diameter ≥2.36 mm) and the total area of air voids in the section image respectively.

The homogeneity of a section image of asphalt mixture cannot represent the actual homogeneity of asphalt mixture; thus, homogeneity coefficient (i.e., *K*) was used to evaluate the homogeneity of asphalt mixture. *K* can be calculated according to Equation (15). The greater the *K*-value, the more serious the segregation of the corresponding asphalt mixture. *K* = 0 represents an ideal state; that is, the aggregates and air voids are evenly distributed in the asphalt mixture.

(15) $$K=\frac{\sum_{i=1}^{n}k_{i}}{N}$$

where *k_i_* is the evaluation index of asphalt mixture homogeneity of the section image numbered *i*, *i* = 1, 2, …, *N*, and *N* is the total number of section images selected.

**Figure 10:**
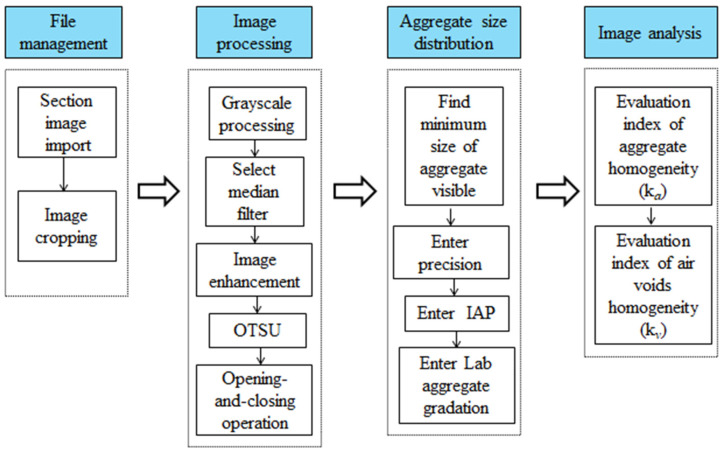
Flow chart of image analysis procedures.

The 2-Dimension Image-based Internal Structure Analysis Method developed by Matlab software, namely 2D-IISAM, could complete the whole image processing process. The detailed image analysis and processing steps were presented in Figure 10.

### 3.4. Rationality of Homogeneity Coefficient

Currently, there are no relevant specifications for testing the homogeneity of an asphalt mixture. Guo et al. proposed to observe the homogeneity of different section images through visual inspection qualitatively, and then analyze whether the homogeneity evaluation index obtained by image processing technique was consistent with the visual inspection result, so as to verify the effectiveness of the homogeneity evaluation index [33]. Since this method was simple and intuitive, this paper adopted this method to preliminarily verify the effectiveness of homogeneity coefficient.

**Figure 11:**
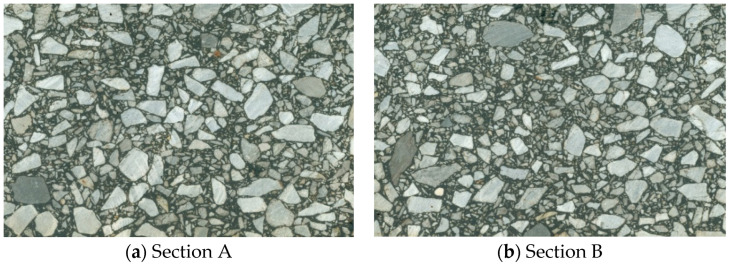
Section image of asphalt mixture specimen.

Two section images of asphalt mixture specimens with obvious differences in homogeneity were selected. Through observation, it could be found that the aggregate distribution in Figure 11a was relatively uniform, and the fine aggregate was concentrated in the middle region in Figure 11b.

The *K* value of a single section calculated based on 2D-IISAM is shown in Table 4. According to the corresponding relationship between actual observation results and *K* value listed in Table 4, it could be basically confirmed that the homogeneity evaluation index proposed in this paper could evaluate the homogeneity of a section image well, that is, it was feasible to use the homogeneity coefficient (i.e., *K*) to characterize the homogeneity of the asphalt mixture specimen.

## 4. Factors Controlling the Homogeneity of Asphalt Mixture

In order to provide theoretical basis for the mixing proportion design process based on the homogeneity of asphalt mixture and further verify the reliability of homogeneity coefficient in the practical application, the effects of nominal maximum aggregate size, aggregate gradation and asphalt content on the homogeneity of asphalt mixtures were analyzed based on 2D-IISAM.

### 4.1. Nominal Maximum Aggregate Size

**Figure 12:**
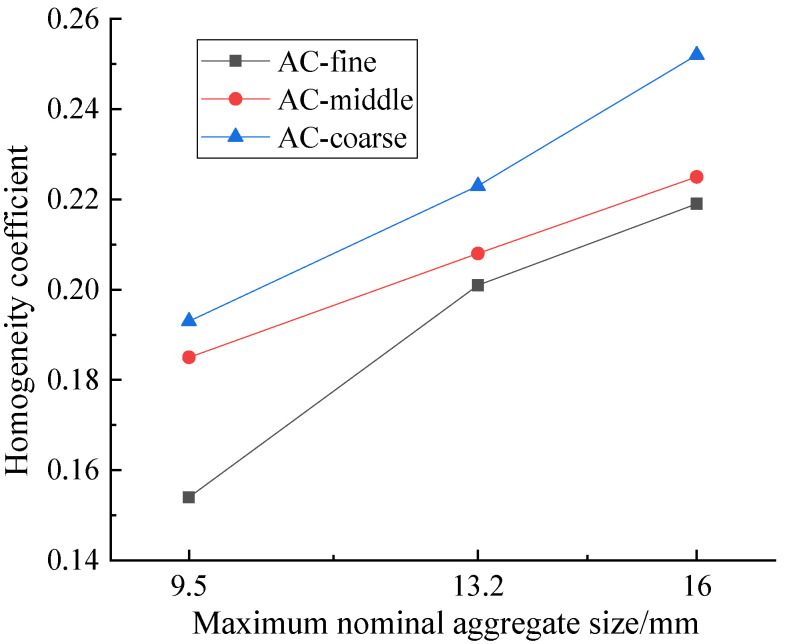
*K* values of asphalt mixture specimens with different maximum aggregate sizes.

To investigate the influence of nominal maximum aggregate size on the homogeneity of asphalt mixtures, the homogeneity of 9 different asphalt mixtures were analyzed. The details of the asphalt mixtures are shown in Table 2, which are numbered 1–9. The homogeneity coefficient of each specimen is shown in Figure 12.

The data listed in Figure 12 shows that with an increase of nominal maximum aggregate size, the homogeneity coefficient increased, indicating that the homogeneity of asphalt mixture became worse. On a good-to-bad scale, the sequence of homogeneities given in Figure 12 can be rated as AC-10, AC-13 and AC-16, which could be attributed to the amount of coarse aggregates in asphalt mixture. For example, the passing percentage of 4.75 mm sieve size in AC-10 middle, AC-13 middle and AC-16 middle were 60%, 53% and 48%, respectively; that is, the mass percentage of coarse aggregates in total aggregate particles were 40%, 47% and 52%, respectively. Compared with coarse aggregate particles, the aggregate with smaller particle size could be more evenly distributed in the asphalt mixture, so AC-10 had better uniformity, vice versa.

For AC-13 mixture, AC-13 coarse mixture with more coarse aggregates and less fine aggregates had the most uneven distribution, while the homogeneity of AC-13 fine mixture with less coarse aggregates and more fine aggregates was best. The more small-size aggregate particles in the mixture, the better the homogeneity of the corresponding asphalt mixture. However, superfine gradation of asphalt mixture would result in the pavement performance being unable to meet the requirements. Therefore, it was inappropriate to blindly select the fine aggregate gradation for the sake of asphalt mixture homogeneity.

### 4.2. Aggregate Gradation

**Figure 13:**
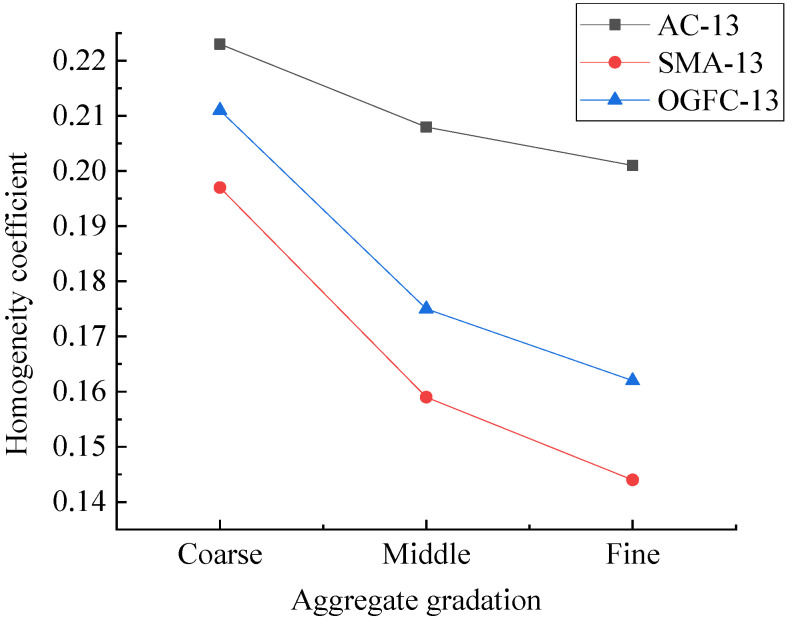
*K* values of asphalt mixture specimens with different gradation types.

In order to explore the influence of aggregate gradation on the homogeneity of an asphalt mixture, the homogeneity coefficients of asphalt mixture specimens numbered 7–15 in Table 2 were analyzed. The results are shown in Figure 13.

Figure 13 reveals that the rank of *K*-value in descending order was AC-13 > OGFC-13 > SMA-13, that is, the homogeneity of asphalt mixture from good to bad was SMA-13, OGFC-13, AC-13. Due to the addition of lignin fiber and large amount of mineral powder in the SMA mixture, the formed mastic asphalt had a high viscosity and could fill the air voids to form a dense skeleton structure, so all phases in the mixture could evenly distributed. Hence, SMA-13 mixture specimen was better than the other two specimens in terms of the homogeneity of the sections. Meanwhile, the OGFC-13 mixture contained fewer aggregate particle size types, so the corresponding homogeneity was also good.

### 4.3. Filler–Binder Ratio

**Figure 14:**
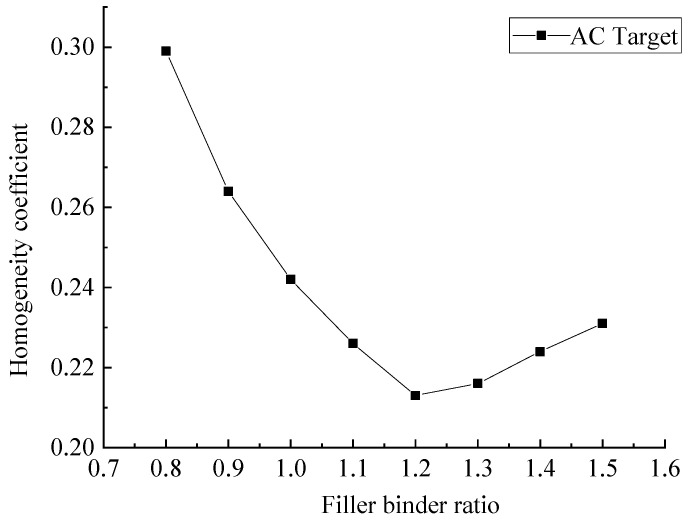
*K* values of asphalt mixture specimens with different filler–binder ratios.

To study the influence of asphalt content on the asphalt mixture homogeneity, the homogeneity coefficients of asphalt mixture specimens numbered 16–23 in Table 2 were analyzed. The results are shown in Figure 14. The filler–binder ratio is the quantity of mineral powder/the quality of asphalt and the corresponding filler–binder ratio is 1.22 under the optimal asphalt content.

Figure 14 shows that with an increase of filler–binder ratio, namely the decrease of asphalt content, the corresponding homogeneity coefficient shows a concave parabola trend. That is, the homogeneity of the asphalt mixture first became better and then became worse, and the corresponding homogeneity of the asphalt mixture was optimal when the asphalt content was near the optimum.

With the increase of filler–binder ratio, the homogeneity of asphalt mixture can be described in three stages:

(1) When the filler–binder ratio was lower (i.e., larger asphalt content), the free asphalt in the asphalt mixture was excessive. At this time, the asphalt not only played a role of binder, but also played a role of lubricant. As a result, the bonding force of asphalt mixture and the interlock force between aggregates decreased. In the compaction process, the displacement between aggregate particles was more likely to occur because there was not enough force to constrain the aggregates, resulting in poor uniformity of asphalt mixture.
(2) When the filler–binder ratio was optimal, the corresponding bonding force and friction resistance of asphalt mixture were moderate. During the compaction process, aggregate particles could be distributed along the optimal trajectory, which made the aggregate particles more evenly distributed in the asphalt mixture.
(3) When the filler–binder ratio was higher, that is, when the asphalt content was insufficient, there was not enough asphalt to form framework asphalt. Meanwhile, the asphalt film thickness was thicker and the friction between aggregates was larger, which would constrain the normal movement trajectory of aggregates and lead to poor uniformity of asphalt mixture.

In summary, there was a certain correlation between the asphalt mixture homogeneity and the filler–binder ratio (i.e., asphalt content). Therefore, the asphalt content must be strictly controlled in the construction process, so as to achieve homogeneous asphalt mixture.

According to the grading range of asphalt mixture stipulated in the national standard of China (JTG F40-2004) and combined with the experimental results of the homogeneity coefficient, the limits for the homogeneity coefficients of different asphalt mixtures are recommended, as shown in Table 5. If the homogeneity coefficient of the mixture is outside this range, it is considered that the homogeneity of the mixture does not meet the requirements. It should be noted that the suggested values of homogeneity coefficient derived in this study are applicable specifically to the compaction method used in this paper and the mixtures studied.

## 5. Conclusions

(1) Based on digital image-processing technique and stereology theory, combined with the virtual aggregate gradation obtained through section images, the distribution characteristics of the inner-structure of components of asphalt mixture can be effectively identified.
(2) The homogeneity coefficient K was proposed to characterize the distribution homogeneity of the asphalt mixture, which could describe the distribution homogeneity of coarse aggregates and air voids comprehensively. For the asphalt mixture with a larger quantity K, it means that the distribution of coarse aggregates and air voids in the asphalt mixture are rather inhomogeneous.
(3) There are strong correlations between the homogeneity coefficient and design factors. With the increase of nominal maximum aggregate size and of the coarseness of aggregate gradation, the homogeneity of asphalt mixture becomes worse. Too much or too little asphalt content is not conducive to the homogeneity of the asphalt mixture. The above results are consistent with previous research rules and further verify the reliability of homogeneity coefficient.
(4) The suggested values of homogeneity coefficient provide theoretical support for the design of asphalt mixture. It should be noted that the suggested values of homogeneity coefficient are applicable specifically to the compaction method used in this paper and the mixtures studied.

## Figures and Tables

**Table 1:** Properties of asphalt binder.

Properties	SBS-Modified Asphalt	High-Viscosity SBS-Modified Asphalt
Penetration (25 °C, 5 s, 100 g)/0.1 mm	71	64
Softening point/°C	57.5	76.5
Ductility (5 °C)/cm	32.4	49.8
Density/(g/cm^3^)	1.021	1.037
Kinematic viscosity (135 °C)/Pa·s	0.81	1.19

**Table 2:** Testing program.

Number	Aggregate Gradation	Nominal Maximum Aggregate Size	Asphalt- Aggregate Ratio (%)	Percent Air Voids	Notes
1	AC Coarse	10	5.0	5.0	Used to analyze the correlation of nominal maximum aggregate size and the homogeneity of asphalt mixture
2	AC Middle	10	5.1	3.7	
3	AC Fine	10	5.2	2.8	
4	AC Coarse	16	4.5	6.1	
5	AC Middle	16	4.7	4.6	
6	AC Fine	16	4.9	3.5	
7	AC Coarse	13	4.7	5.5	
8	AC Middle	13	4.9	3.9	
9	AC Fine	13	5.1	2.9	
10	SMA Coarse	13	5.7	5.0	Used to analyze the correlation of aggregate gradation and the homogeneity of asphalt mixture
11	SMA Middle	13	5.9	3.5	
12	SMA Fine	13	6.1	2.6	
13	OGFC Coarse	13	4.5	22.9	
14	OGFC Middle	13	4.7	20.4	
15	OGFC Fine	13	4.9	18.7	
16	AC Target	13	7.5	1.0	Used to analyze the correlation of asphalt content and the homogeneity of asphalt mixture
17	AC Target	13	6.67	1.9	
18	AC Target	13	6.0	2.7	
19	AC Target	13	5.45	3.2	
20	AC Target	13	5.0	3.5	
21	AC Target	13	4.62	4.2	
22	AC Target	13	4.29	5.7	
23	AC Target	13	4.0	7.3	

**Table 3:** Aggregate gradation.

Gradation Type	19	16	13.2	9.5	4.75	2.36	1.18	0.6	0.3	0.15	0.075
AC-10 Coarse	100	100	100	91	49	34	23	16	11	7	5
AC-10 Middle	100	100	100	95	60	44	32	23	16	11	6
AC-10 Fine	100	100	100	99	71	54	41	29	21	15	7
AC-13 Coarse	100	100	91	70	42	28	18	13	9	6	5
AC-13 Middle	100	100	95	77	53	37	27	19	14	10	6
AC-13 Fine	100	100	99	83	64	46	35	25	18	14	7
AC-13 Target	100	100	96	78	44	33	23	17	11	9	6
AC-16 Coarse	100	91	78	63	38	24	16	11	9	6	5
AC-16 Middle	100	95	84	70	48	34	25	18	13	10	6
AC-16 Fine	100	99	90	77	58	44	33	24	16	13	7
SMA-13 Coarse	100	100	91	53	22	16	15	13	11	10	8
SMA-13 Middle	100	100	95	63	27	21	19	16	13	12	10
SMA-13 Fine	100	100	99	72	32	25	23	19	15	14	12
OGFC-13 Coarse	100	100	91	62	14	11	7	5	4	4	3
OGFC-13 Middle	100	100	95	70	21	16	12	10	8	6	4
OGFC-13 Fine	100	100	99	78	28	21	17	14	11	7	5

**Table 4:** Results of homogeneity evaluation index.

Section Number	Actual Observation Result	*k*
a	The asphalt mixture is evenly distributed	0.201
b	Fine aggregates are concentrated in the middle region	0.247
Ideal section image	--	0

**Table 5:** Suggested value of homogeneity coefficient.

Type of Asphalt Mixture	Suggested Value
AC-10	[0.15,0.20]
AC-16	[0.21,0.26]
AC-13	[0.19,0.23]
SMA-13	[0.14,0.20]
OGFC-13	[0.16,0.22]

**Note:** 10/13/16 refers to the nominal maximum aggregate size.

## Data Availability

Not applicable.

## References

[1] Ma T., Wang Z., Zhao Y., Huang X. (2011). Evaluation of dispersive performance of asphalt mixture during mixing of hot in-place recycling. J. Harbin Inst. Technol..

[2] Peng Y., Xu X. (2013). Numerical analysis of effect of aggregate distribution on splitting strength of asphalt mixtures. J. Zhejiang Univ..

[3] Chen C., Williams R.C., Ahmed E.T., Lee H.D., Schram S. (2013). Quality control/quality assurance testing for longitudinal joint density and segregation of asphalt mixtures. Constr. Build. Mater..

[4] Airey G.D., Collop A.C. (2016). Mechanical and structural assessment of laboratory-and field-compacted asphalt mixtures. Int. J. Pavement Eng..

[5] Cao W., Liu S., Xue Z., Chen L., Zhang Z. (2021). Laboratory method to characterize coarse aggregate segregation for HMA. J. Mater. Civil Eng..

[6] Yu H., Yang M., Qian G., Cai J., Zhou H., Fu X. (2021). Gradation segregation characteristic and its impact on performance of asphalt mixture. J. Mater. Civil Eng..

[7] Chun S., Kim K., Park B., Greene J. (2018). Evaluation of the effect of segregation on coarse aggregate structure and rutting potential of asphalt mixtures using Dominant Aggregate Size Range (DASR) approach. KSCE J. Civ. Eng..

[8] Zhang K., Sun P., Li L., Zhao Y., Zhao Y., Zhang Z. (2021). A novel evaluation method of aggregate distribution homogeneity for asphalt pavement based on the characteristics of texture structure. Constr. Build. Mater..

[9] Stroup-Gardiner M., Law M., Nesmith C. (2000). Using infrared thermography to detect and measure segregation in hot mix asphalt pavements. Int. J. Pavement Eng..

[10] Wu J., Romero P. (2004). Analysis of multivariate models for evaluating segregation in hot-mix asphalt pavements. Transp. Res. Rec..

[11] Hu C., Wang D., Thyagarajan S. (2010). Homogeneity evaluation of compacted asphalt mixture based on X-ray computed tomography. J. Chang’an Univ..

[12] Sefidmazgi N.R., Tashman L., Bahia H.U. (2012). Internal structure characterization of asphalt mixtures for rutting performance using imaging analysis. Road Mater. Pavement Des..

[13] Coenen A.R., Kutay M.E., Sefidmazgi N.R., Bahia H.U. (2012). Aggregate structure characterisation of asphalt mixtures using two-dimensional image analysis. Road Mater. Pavement Des..

[14] Bruno L., Parla G., Celauro C. (2012). Image analysis for detecting aggregate gradation in asphalt mixture from planar images. Constr. Build. Mater..

[15] Zhang K., Zhang Z., Luo Y., Huang S. (2017). Accurate detection and evaluation method for aggregate distribution uniformity of asphalt pavement. Constr. Build. Mater..

[16] Hunter A., Airey G., Collop A. (2004). Aggregate orientation and segregation in laboratory compacted asphalt samples. Transp. Res. Rec..

[17] Wu W., Li Z., Zhang X. (2009). Evaluation of asphalt mixture homogeneity with digital image processing technique. J. Jilin Univ..

[18] Thyagarajan S., Tashman L., Masad E., Bayomy F. (2010). The heterogeneity and mechanical response of hot mix asphalt laboratory specimens. Int. J. Pavement Eng..

[19] Zhang J., Liu H., Wang P., Pei J., Bao D., Jin L. (2017). Evaluation of aggregate gradation and distributing homogeneity based on the images of asphalt mixture. Road Mater. Pavement Des..

[20] Li X., Lv X., Zhou Y., Diab A., Chen Y., Cui Z., You Z. (2020). Homogeneity evaluation of hot in-place recycling asphalt mixture using digital image processing technique. J. Clean. Prod..

[21] Peng Y., Sun L. (2009). Towards an Index of Asphalt Mixture Homogeneity. Road Mater. Pavement Des..

[22] Peng Y., Sun L. (2014). Horizontal homogeneity in laboratory-compacted asphalt specimens. Road Mater. Pavement Des..

[23] Peng Y., Harvey J., Sun L. (2017). Three-dimensional discrete-element modeling of aggregate homogeneity influence on indirect tensile strength of asphalt mixtures. J. Mater. Civil Eng..

[24] Peng Y., Sun L. (2017). Aggregate distribution influence on the indirect tensile test of asphalt mixtures using the discrete element method. Int. J. Pavement Eng..

[25] (2004). Technical Specifications for Construction of Highway Pavements.

[26] (2011). Standard Test Methods of Bitumen and Bituminous Mixtures for Highway Engineering.

[27] Wu W., Wang D., Zhang X., Li Z. (2009). Stereology method of estimation gradation of asphalt mixtures. China J. Highw. Transp..

[28] Sefidmazgi N.R. (2014). Hot Mix Asphalt Design to Optimize Construction and Rutting Performance Properties. Ph.D. Thesis.

[29] Otsu N. (2007). A threshold selection method from gray-level histograms. IEEE Trans. Syst. Man Cybern..

[30] Sha A., Wang C., Sun C. (2010). An image-based mineral gradation measurement method of asphalt mixture. J. Chang’an Univ..

[31] Coenen A.R. (2011). Image Analysis of Aggregate Structure Parameters as Performance Indicators of Rutting Resistance. Ph.D. Thesis.

[32] Guo N., You Z., Tan Y., Zhao Y., Jing H. (2017). Evaluation method for homogeneity of asphalt mixtures based on CT technique. China J. Highw. Transp..

[33] Guo N., Wang C., Zhao Y., Tan Y., You Z. (2016). Homogeneity of internal structure of asphalt mixtures based on density of inclusions. Eng. Mech..

